# How common is remission in juvenile idiopathic arthritis: A systematic review

**DOI:** 10.1016/j.semarthrit.2017.05.007

**Published:** 2017-12

**Authors:** Stephanie J.W. Shoop-Worrall, Lianne Kearsley-Fleet, Wendy Thomson, Suzanne M.M. Verstappen, Kimme L. Hyrich

**Affiliations:** a Division of Musculoskeletal and Dermatological Sciences, Faculty of Biology, Medicine and Health, Arthritis Research UK Centre for Epidemiology, Centre for Musculoskeletal Research, Institute of Inflammation and Repair, University of Manchester, Manchester, UK; b NIHR Manchester Musculoskeletal Biomedical Research Unit, Central Manchester University Hospitals NHS Foundation Trust and University of Manchester Partnership, Manchester, UK; c Division of Musculoskeletal and Dermatological Sciences, Faculty of Biology, Medicine and Health, Arthritis Research UK Centre for Genetics and Genomics, Centre for Musculoskeletal Research, Institute of Inflammation and Repair, University of Manchester, Manchester, UK

**Keywords:** PGA, physician’s global assessment, PGE, parental global evaluation, ESR, erythrocyte sedimentation rate, JADAS, Juvenile Arthritis Disease Activity Score, cJADAS, clinical JADAS, PICO, Patient Intervention Comparison Outcome, JRA, juvenile rheumatoid arthritis, JCA, juvenile chronic arthritis, QA, quality assessment, EULAR, European League against Rheumatism, ACR, American College of Rheumatology, RF, rheumatoid factor, ERA, enthesitis-related arthritis, PsA, psoriatic arthritis, Juvenile idiopathic arthritis, Remission, Clinically inactive disease, Paediatric rheumatology, Systematic review

## Abstract

**Objectives:**

The ideal goal of treatment for juvenile idiopathic arthritis (JIA) is disease remission. However, many sets of remission criteria have been developed and no systematic review of remission in JIA exists.

The current systematic review investigated (1) how remission has been defined across JIA clinical cohorts and (2) the frequency of remission overall and within disease categories.

**Methods:**

Studies using prospective inception cohorts published after 1972 were selected if they estimated remission in cohorts of ≥50 patients. Articles focusing on specific medical interventions, not defining remission clearly or not reporting disease duration at remission assessment were excluded. Studies were selected from Medline, Embase, PubMed and bibliographies of selected articles. Risks of selection, missing outcome data and outcome reporting biases were assessed.

**Results:**

Within 17 studies reviewed, 88% had majority female participants and patient disease duration ranged from 0.5 to 17 years. Thirteen sets of criteria for clinically inactive disease and remission were identified. Uptake of Wallace’s preliminary criteria was good in studies recruiting or following patients after their publication (78%).

Remission frequencies increased with longer disease duration from 7% within 1.5 years to 47% by 10 years following diagnosis. Patients with persistent oligoarticular and rheumatoid-factor positive polyarticular JIA were most and least likely to achieve remission, respectively.

**Conclusions:**

Achievement of remission increased with longer disease duration, but many patients remain in active disease, even in contemporary cohorts. Multiple sets of outcome criteria limited comparability between studies.

## Introduction

Juvenile idiopathic arthritis (JIA) is the most common chronic rheumatic disease in childhood [1]. The presentations, treatments and outcomes are variable across this heterogeneous disease, but the main goal for all patients is disease remission, in order to prevent or reduce the long-term pathologies, such as pain and functional disability [2].

Various sets of remission criteria have been applied across clinical cohorts and in clinical trials, although most aim to identify a state of minimal or absent disease activity. Composite remission criteria often include one or more measures across the core JIA outcome variables including active joint counts, global assessment score of disease activity by physician’s (PGA) or parents (PGE) and erythrocyte sedimentation rate (ESR) [3], with additional criteria including activity of systemic features for children with systemic JIA [4], [5], presence of ocular inflammation in uveitis and length of morning stiffness [4]. The first validated composite criteria for assessing this disease state were Wallace’s preliminary criteria for clinically inactive disease and remission in JIA (2004) [5], which divides remission into three following distinct states: clinically inactive disease (i.e., no apparent disease activity at a single time point), remission on medication (i.e., clinically inactive disease maintained for at least 6 months whilst taking anti-rheumatic and/or anti-uveitis medication) and remission off medication (i.e., clinically inactive disease maintained for at least 12 months without medication) [5]. Other recent sets of criteria validated for use in JIA include cut-offs of the Juvenile Arthritis Disease Activity Score (JADAS) [6] and clinical JADAS (cJADAS) [7] to represent states of clinically inactive disease and remission.

The literature on the frequency of remission in JIA has never been reviewed systematically. Knowledge of the frequency of remission across clinical cohorts would provide an insight into the past and current disease course of JIA, both overall and within specific disease categories. Three narrative reviews [8], [9], [10] were published prior to or in the immediate years after the publication of Wallace’s preliminary criteria [5]. Ravelli reported the frequency of remission in JIA at between 35% and 61% [10], with Adib et al. [8] estimating this frequency at between 33% and 56%. In addition to pre-dating uptake of the validated sets of remission criteria for JIA, these reviews focused on cohorts largely recruited before the introduction of biologic therapies for JIA at the turn of the century [11]. Thus, the uptake of newer set of criteria for remission and the remission rates in cohorts with access to biologic therapies have not been described. In 2010, Shenoi and Wallace [12] reviewed six studies that had utilised the Wallace’s preliminary criteria. However, studies reviewed were largely retrospective in design, therefore likely excluding a portion of children with milder disease features. The only prospective study reviewed was in children with systemic JIA, therefore the generalisability of the review to other recent JIA cohorts was limited. The review also did not consider how remission frequencies are affected with increasing disease duration.

The aims of this systematic review were therefore to: (i) investigate how remission has been defined across JIA clinical cohorts and (ii) describe the frequency of remission in cohorts of JIA overall and within individual disease categories.

## Methods

### Search strategy

Medline, Embase and PubMed databases were searched from January 1972 to March 2015 by author SJWS, using patient intervention comparison outcome (PICO) methodology to build the following strategy: (P) patients with juvenile rheumatoid arthritis (JRA) [13], juvenile chronic arthritis (JCA) [14] or JIA [15], (I) no specified intervention, (C) not applicable and dropped from search design and (O) remission or clinically inactive disease. The study was built and reported according to PRISMA guidelines [16]. Patients of all ages and disease durations were included to summarise short and long-term remission frequencies. Synonyms of each PICO were applied (full search terms and hits detailed in Supplementary Table 1). Where articles were selected for inclusion, their bibliographies were also screened for further relevant articles.

### Inclusion and exclusion criteria

The inclusion criteria for studies included the following: (i) reported the frequency or proportion of patients in remission, (ii) utilised patients from inception cohorts with at least partial prospective data collection, (iii) were available in English, (iv) included at least 50 patients, (v) did not focus on remission following a specific medical intervention, (vi) did not recruit a specific group of patients based on investigations (e.g., imaging) or location of affected joints and (vii) included information on disease duration at time of remission assessment. Studies using the same patient population were included if reporting outcomes at different follow-up intervals or used different sets of outcome criteria.

Case reports, clinical trials and non-original research articles were excluded. Article titles and abstracts were independently reviewed by two reviewers S.J.W.S. and L.K.F., after which an agreed list of full text articles were screened independently. Full texts were accessed where abstracts suggested the study might meet the inclusion criteria or did not contain enough information to assess relevance. Where studies from the same population reported the same outcome over the same follow-up period, the publication with the most detailed information on remission (e.g., used secondary criteria or time point) was selected and the other(s) excluded (*n* = 2 [17], [18]). Where there was disagreement or uncertainty at any stage, a third reviewer K.L.H. adjudicated.

### Quality assessment

Risk of bias within selected articles was assessed using a modified version of Pasma et al. [19] quality assessment (QA) tool (Supplementary Table 2). “Essential questions” assessed risk of bias associated with the patient sampling method, disclosure of differences between consents and refusals, missing outcome data and outcome definition reporting. Non-relevant questions from the Pasma tool were dropped. In addition, a question on missing data bias, adapted from the Cochrane Collaboration tool for assessing risk of bias [20], was added.

For each of eight bias categories, one point was scored where evidence of avoiding or controlling for the relevant bias was evident. Articles that scored at least three out of the four on the “essential” questions and at least five out of the eight in total were considered to be of high quality. Since studies were observational in design and did not focus on specific medical interventions, risk of bias across, rather than within, studies was not assessed.

### Description and evidence synthesis

Information on study location, follow-up period, outcome sets of criteria and the frequency of remission were extracted independently. In addition, participant sampling frames were extracted.

Sets of criteria for clinically inactive disease or remission were identified and classified as “validated” or “investigator-defined.” If validated sets of criteria had been altered, this was noted but classed as “investigator-defined.” For the purposes of the review, outcomes were classed as “clinically inactive disease,” where no evidence of disease activity was apparent at a single time point, or “remission,” whereby clinically inactive disease had been maintained off medication and/or for a specific length of time at assessment.

Estimates were compared between studies that assessed “current remission” at the end of follow-up. Additionally, estimates were compared between those assessing “ever remission” throughout follow-up. Both clinically inactive disease and remission estimates were then compared between validated and investigator-defined sets of criteria and with increasing disease duration. Finally, the ranges of clinically inactive disease and remission estimates across different ILAR categories were synthesised.

## Results

**Fig. 1:**
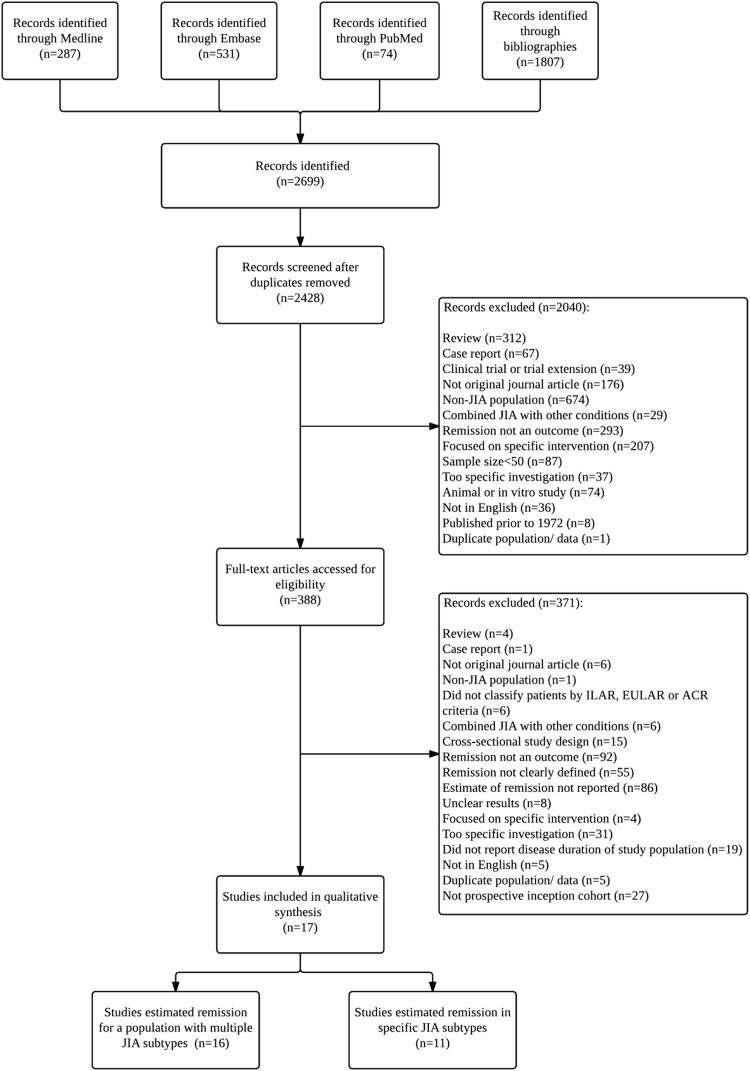
Number of articles accessed and reviewed to explore the frequency of remission in JIA. “Too specific investigation” refers to inclusion criterion 6 in the text.

Of 2428 unique articles identified, 17 were selected for inclusion to the systematic review (Fig. 1). Of included studies, 16 reported the frequency of remission for the cohort overall and 11 for specific disease categories (Fig. 1). Disease categories classified under European League against Rheumatism (EULAR) [14] or American College of Rheumatology (ACR) [13] criteria were pooled with corresponding categories in the ILAR criteria [15] (e.g., pauciarticular juvenile arthritis and oligoarticular JIA).

### Risk of bias in selected articles

Overall, study quality was moderate with only 9/17 (53%) articles fulfilling the criteria for “high quality” on the QA tool (Supplementary Table 2). Of the four key components, 15/17 (88%) reduced the risk of selection bias through appropriate sampling methods but only 11/17 (65%) reduced this risk through comparisons of patients consenting and not consenting to participate. In addition, 16/17 (94%) applied reproducible remission criteria. However, only 3/17 (18%) reduced the risk of missing data bias through not having missing outcome data or applying appropriate methodologies to manage these (Supplementary Table 3).

### Study characteristics

The majority of patient populations were located in Europe (*n* = 12, 71%) (Table). Cohorts were reasonably large, with the majority recruiting patients from multiple clinics (*n* = 13, 76%) and only 3/17 (18%) following fewer than 100 patients. The most recent classification system (ILAR) was used in most studies (*n* = 10, 59%), although of the remaining seven, four were published prior to the publication of this set of criteria (Table).

**Table:** Demographic and disease information of articles reviewed

Author	Country	Sample size	Juvenile arthritis classification	Percentage female (%)	Percentage by category (%)							
					Systemic	Oligo	RF− Poly	RF+ Poly	Total Poly	ERA	PsA	Undiff.
*Multi centre*												
Guzman et al. [23]	Canada	1104	ILAR	64	7	38	21	4	–	14	6	10
Berntson et al. [24]	From the Nordic JIA database	192	ILAR	69	5	51	21	1	–	8	1	15
Berntson et al. [25]	Scandinavia and Finland	410	ILAR	66	4	47	18	1	–	11	3	15
Shen et al. [26]	Taiwan	195	ILAR	45	19	23	12	5	–	37	2	3
Bertilsson et al. [27]	Sweden	132	EULAR	64	7	64	–	–	22	5	2	0
Shen et al. [21]	Taiwan	58	ILAR	41.4	16	17	–	–	28	40	–	–
Bertilsson et al. [28]	Sweden	128	EULAR	64	4.7	64	–	–	27	3.1	1.6	–
Nordal et al. [29]	Scandinavia and Finland	440	ILAR	66	4	51	21	1	–	8	1	14
Oen et al. [30]	Canada	356	ILAR	66	7	41	20	4	–	10	7	12
Berntson et al. [31]	Denmark, Norway, Sweden and Finland	312	ILAR	72		55	25	1.9	–	3.8	2.2	12
Gäre et al. [32]	Sweden	124	EULAR	65	3.2	58	–	–	29	4.8	4.8	–
Gäre et al. [33]	Sweden	124	EULAR	65	3.2	58	–	–	29	4.8	4.8	–
Gäre et al. [34]	Sweden	Two groups: G1: 121	EULAR	65 (G1)	2 (G1)	31 (G1)	–	–	48 (G1)	15 (G1)	5 (G1)	–
		G2: 125		64 (G2)	4 (G2)	46 (G2)			40 (G2)	5 (G2)	5 (G2)	
*Single centre*												
Padeh et al. [35]	Israel	75	ILAR	65	8	68	11	–	–	4	4	5
Selvaag et al. [36]	Norway	197	ACR	61	7	56	28	3	–	4	3	–
Kotaniemi et al. [22]	Finland	372	ILAR	66	–	73	27	–	–	–	–	–
Flatø et al. [37]	Norway	72	ACR	54	6	44	–	–	24	17	10	–

Studies are listed first by whether cohorts are multi/single centre, by year of publication and finally according to sample size. Disease categories: Oligo, oligoarticular; Poly, polyarticular; RF, rheumatoid factor; total poly, polyarticular where RF status was not determined; ERA, enthesitis-related; PsA, psoriatic; Undiff., undifferentiated JIA.

In the majority of patient populations, there were greater numbers of females than males (*n* = 15). Oligoarticular JIA was frequently the most common disease category (*n* = 14, 82%) and ranged from 17% [21] to 73% [22] of cohorts across all studies. Two popoulations from Taiwan comprised greater males than females, with enthesitis-related JIA the most common ILAR category [21], [26] (Table).

### The frequency of remission in selected studies

#### Sets of criteria for clinically inactive disease and remission used

Across 17 studies, 13 different sets of criteria for remission or clinically inactive disease were identified. The majority of these were investigator-defined. Only seven studies applied previously validated sets of criteria for remission in JIA in full: all applied Wallace’s Preliminary Criteria (Supplementary Table 4).

#### Point prevalence estimates using Wallace’s preliminary criteria

Of studies quantifying current clinically inactive disease and remission across the entire cohorts, seven of nine (78%) that followed at least part of their patient cohorts after their publication used Wallace’s preliminary criteria.

**Fig. 2:**
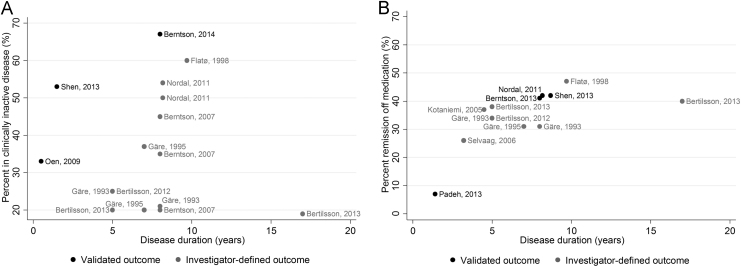
Percentage of patients with JIA in current (A) clinically inactive disease or (B) remission off medication across the literature. Point estimates are stratified based on whether outcome definitions were validated or investigator-defined. Where studies are listed multiple times, multiple sets of outcome criteria have been utilised (Supplementary Table 4).

The prevalence of current clinically inactive disease using Wallace’s preliminary criteria increased between 33% [30] at 6 months to 67% [24] at 8 years (Fig. 2A). Similarly, the prevalence of current remission off medication using Wallace’s preliminary criteria increased from 7% at mean 1.5 years (±0.5 years) [35] to 42% at median 8 years (IQR: 7–12 years) [29] (Fig. 2B). Only two studies applied the criteria for remission on medication. At 9% and 15% after approximately 8 years of disease (IQR: 6–13 years), these estimates were substantially lower than the estimates of remission off medication after similar follow-up [26], [29] (Fig. 2B and Supplementary Table 4).

#### Point prevalence estimates using investigator-defined sets of remission criteria

Across cohorts, the prevalence of clinically inactive disease using investigator-defined sets of criteria varied widely between 19% [27] and 60% [37]. These estimates did not seem to be associated with disease duration (Fig. 2A). However, definitions of clinically inactive disease and remission were not always nested and many studies did not include children who were in remission into the estimates of clinically inactive disease [27], [28], [29], [32], [34]. There appeared to be a slight increase in remission achievement, using investigator-defined sets of criteria, over time from 26% [36] to between 40% and 50% [27], [37] over a period of at least ten years of disease (Fig. 2B).

#### Point prevalence estimates of current remission across ILAR categories

Patients with persistent oligoarticular JIA appeared to achieve clinically inactive disease and remission most frequently when compared to other categories, with remission off medication estimates ranging from 39% at mean 3 years (±0.4 years) [36] to 66% at median 9 years (IQR: 6–13 years) [26]. In contrast, patients with RF-positive polyarticular disease and enthesitis-related JIA appeared to achieve these states the least often, with 2/5 studies on the former and 3/8 studies on the latter reporting that none of these patients achieved remission at follow-up [27], [28], [29], [36]. Estimates from patients with systemic disease exhibited the largest variation, ranging from 0% [37] to 100% [32] in some form of clinically inactive disease or remission (Supplementary Table 4).

### Estimates of ever having achieved clinically inactive disease and remission

Only three studies investigated the cumulative percentage of ever achieving clinically inactive disease or ever achieving remission over time [23], [26], [37]. Guzman et al. estimated the ever achievement of clinically inactive disease to range from 45% within 1 year to 95% within 5 years. The estimates for ever remission off medication from this article ranged from 4% within 2 years to 41% within 5 years following diagnosis with other estimates from longer follow-ups at 45% within median 9 years (IQR: 6–13 years) [26] and 81% within mean 10 years (±2 years) [37]. Guzman et al. [23] reported that patients with oligoarticular JIA achieved highest achievement and RF-positive polyarticular JIA the lowest achievement of remission, with 0% of this latter category ever achieving remission off medication within the first 5 years of disease.

## Discussion

This systematic review aimed to quantify the frequency of clinically inactive disease and remission in patients with juvenile-onset arthritis. Patient populations spanned four continents, although the majority of the included studies originated in Scandinavia. The achievement of remission increased with increasing disease duration, although after over a decade of disease, fewer than half of patients have achieved this state. Large variations were identified in outcome criteria and resulting outcome frequencies.

With 13 sets of criteria for either clinically inactive disease or remission applied in 17 studies, comparability between estimates was compromised. Seven studies used a published set of criteria: Wallace’s preliminary criteria, which have undergone internal validation [38]. It is likely that, particularly for earlier cohorts, all necessary measures were not collected from cohort inception to enable the use of published sets of criteria such as Wallace’s preliminary criteria. In addition, since blood tests are not mandatory for all patients in real-world observational cohorts, investigators frequently did not have all outcome data, such as ESR, to apply the full validated set of criteria. This is illustrated in that a maximum of three components of Wallace’s preliminary criteria were used in any of the investigator-defined outcomes. However, the frequent altering of validated sets of criteria in a non-uniform manner means both that validated sets of criteria are no longer being used and that remission rates cannot be directly compared or pooled to attain an average. A consensus on how to apply published sets of criteria should be reached in order to standardise outcome assessment across clinical cohorts.

Whilst clinically inactive disease estimates were extremely variable in studies using investigator-defined sets of outcome criteria, a clearer trend was evident in cohorts using Wallace’s preliminary criteria ranging from 33% at 6 months [30] to 67% at 8 years [24]. The variation in estimates from studies using investigator-defined criteria sets likely stems from a combination of different outcome definitions and the non-inclusion of patients in remission into clinically inactive disease estimates in many of the studies [27], [28], [29], [32], [34]. By not combining children who have achieved clinically inactive disease and remission in the former estimates, these studies underestimate the achievement of clinically inactive disease. Across all cohorts, a greater number of patients achieved remission with increasing disease duration. This trend likely reflects initial disease control for patients responsive to first-line therapies and differential delays to effective therapies in patients with refractory disease. These remission estimates ranged from under 7% [35] early in the disease course to between 40% and 50% after 10–20 years of disease [27], [37]. However, these latter reports likely underestimate remission due to the lack of biologic therapies for these older cohorts and likely attrition of patients due to low disease activity earlier in the disease course.

The clearer trend in remission estimates compared with those for clinically inactive disease likely stems from more similar sets of outcome criteria and the relative ease of capturing data on the former, since clinically inactive disease is a transient state and may last for only a short period before relapse occurs [38]. Thus, short periods of clinically inactive disease may not coincide with a study visit. Indeed, in three of the studies reviewed by Shenoi and Wallace [12], patients achieved clinically inactive disease multiple times over the study periods, but may not have retained this disease state long enough to be classified as in remission. Future studies should, therefore, attempt to measure “ever” clinically inactive disease and remission to capture the changing disease processes of JIA.

Estimates of remission on and off medication differed substantially, particularly when Wallace’s preliminary criteria had been used. Whilst remission on medication is intended as an intermediate between clinically inactive disease and remission off medication [5], these estimates fell below those of remission off medication in the current review. Since there are currently no published guidelines on when and how to discontinue treatment in JIA once clinically inactive disease has been achieved [39], it is likely that different tapering strategies existed across patient populations. Remission on medication cannot be measured if medication is discontinued at, or shortly after, achievement of clinically inactive disease. It is likely, therefore, that remission on medication can be captured in children with more severe disease, who may receive longer-term medication, potentially to avoid relapse [40]. In accordance, Shen et al. [26] reported rates of remission on medication that exceeded that off medication only in the more severe RF+ polyarticular category. An alternative definition strategy was demonstrated by Flatø et al. [41], who altered remission on medication on Wallace’s preliminary criteria [5] to allow the maintenance of clinically inactive disease on or off medications for 6 months. By removing the requirement to be off medication, their estimate of remission on medication exceeded that of remission off medication and likely was a more representative intermediate between the two states.

Similar to all three previous reviews [8], [10], [12], this review corroborated that patients with persistent oligoarticular disease seem to have the most favourable disease course. However, patients with enthesitis-related JIA appear to have relatively poor prognosis together with, in accordance with previous accounts [8], [10], [12], patients with RF+ polyarthritis. Those with systemic JIA were reported to have the largest variation in achievement of clinically inactive disease and remission, ranging from 0% [37] to 100% [32], irrespective of time followed. This large variation likely stems from the different outcome definitions that capture variable elements of systemic disease, rather than any particularly diverse outcomes experienced by patients with systemic JIA within different studies.

### Strengths and limitations

This was the first review of remission in JIA conducted systematically. That all published estimates from relevant inception cohorts were included lends these estimates far more generalisable to the general population of patients with JIA than any from previous reviews. A total of 12 years have passed since the introduction of Wallace’s preliminary criteria [5] and five since the introduction of the ACR 2011 criteria [4], which allows assessment of definition uptake within clinical studies. No study has so far published outcomes described using the recently proposed JADAS clinically inactive disease cut-offs [6], [7]. Future work should encourage the uptake of these more novel sets of criteria to assess their performance in real-world datasets.

Limitations of the current review related to study quality with regards to selection and missing data biases. This review highlights that few inception cohorts have reported on the outcome of disease remission or clinically inactive disease. This may reflect a paucity of studies, or that existing studies either do not have the available data or have not been recruiting children long enough to report these outcomes. Heterogeneity between studies, which have been published hindered the comparability of data extracted. It is noted that many studies were from Scandinavia and therefore the results may not be directly applicable to countries with significantly different health care systems or access to treatments. Further research needs to assess the outcomes in other populations not included in this review. In addition, the vast majority of studies did not deal with missing outcome data appropriately and as evidenced by Fantini et al. [42], patients lost to follow-up are more likely to be in remission than those who continue presenting to clinic. By excluding patients with incomplete data at baseline or not imputing their outcome data using appropriate methods, the frequencies of remission are likely to be underestimated in these studies. In addition, approximately a third of studies either did not have at least 80% participation or did not compare patients that consented and those that refused to participate. However, these selection biases were minimised by only including inception cohorts in the current review.

Few studies reported the cumulative achievement of clinically inactive disease or remission. Since the disease course in JIA is one of remitting and relapse [43], estimates of “ever” rather than “current” remission may give a better overall picture of disease activity in affected children. In addition, the number of repeated periods in remission or length of sustained remission over study follow-up would ideally be captured. In order to explore if the current estimates are accurate and to increase the knowledge of the patterns of remission in JIA, further work should explore these outcomes. In addition, the changes in achievement of these states could not be assessed between pre- and post-biologics eras. Recruitment to many of the studies spanned the introduction of biologic therapies and the uptake of more aggressive treatment strategies. It was therefore unclear which patients in each study had been exposed to these new strategies and which had not been. This review could, therefore, not associate specific therapies with achievement of clinically inactive disease or remission, particularly since wider and earlier use of methotrexate in the same period as the introduction of biologic therapies [44] may also have influenced results.

The largest limitation of the review was the inability to pool or directly compare results. This stemmed from the vast number of sets of clinically inactive disease and remission criteria. The number of criteria sets continues to increase and it is not yet clear which should be designated, if any, as main set of outcome criteria for use in observational research in JIA. Since there is no “gold-standard” for remission in JIA, current published criteria sets have been validated against different surrogate measures. It is, therefore, unclear if the same construct is being assessed across sets of criteria. Further work should assess the degree of overlap between these sets of criteria through comparisons in a single population at a common time point.

## Conflicts of Interest

The authors declare no conflicts of interest.

## Conclusions

In this first systematic review of remission in JIA, the frequency of current remission increased with increasing disease duration from 7% at 18 months to around 40% after at least 10 years. Large variation in estimates existed, largely driven by differences in the 13 sets of outcome criteria utilised. Patients with persistent oligoarticular disease had high achievement of remission with those in RF+ polyarticular category the lowest.
