# Supplementary material for: How common is remission in juvenile idiopathic arthritis: A systematic review
Source: Semin Arthritis Rheum. 2017 Dec;47(3):331–7. doi: 10.1016/j.semarthrit.2017.05.007 (PMC5687936; doi:10.1016/j.semarthrit.2017.05.007)
Supplement: Supplementary file 1 — Supplementary material [file mmc1.docx]

### SUPPLEMENTARY MATERIALS

No review protocol has been previously published.

Supplementary Table 1. Search strategy and results for each database

| # | Search History | Results |
| --- | --- | --- |
| Medline | | |
| 1 | exp ARTHRITIS, JUVENILE/ cl, di, dt, ep, ge, im, pc, su, th. | 5894 |
| 2 | JIA | 1880 |
| 3 | CHILD* or ADOLESCENT | 2712565 |
| 4 | ARTHRITIS/ cl, di, dt, ep, ge, pc, rh, su, th | 12146 |
| 5 | 3 and 4 | 1790 |
| 6 | 1 or 2 or 5 | 8235 |
| 7 | exp REMISSION, INDUCTION | 32934 |
| 8 | exp REMISSION, SPONTANEOUS/ bl, ci, di, dt, ep, im, pp, rt, th | 232 |
| 9 | REMISSION | 110391 |
| 10 | DISEASE ACTIVITY | 25038 |
| 11 | LOW or MINIMAL or ABSEN* or QUIESCE* | 2139027 |
| 12 | 10 adj3 11 | 753 |
| 13 | DISEASE OUTCOME* | 5655 |
| 14 | INACTIVE DISEASE | 1173 |
| 15 | ACCEPTABLE SYMPTOM STATE | 47 |
| 16 | 7 or 8 or 9 or 12 or 13 or 14 or 15 | 118118 |
| 17 | 6 and 16 | 546 |
| 18 | Limit 17 to (CLINICAL TRIAL, ALL or RANDOMIZED CONTROLLED TRIAL) | 74 |
| 19 | Limit 17 to CASE REPORTS | 76 |
| 20 | Limit 17 to (CLINICAL CONFERENCE or CONGRESSES) | 1 |
| 21 | Limit 17 to (META ANALYSIS or REVIEW or SCIENTIFIC INTEGRITY REVIEW or SYSTEMATIC REVIEWS) | 90 |
| 22 | 17 not (18 or 19 or 20 or 21) | 318 |
| 23 | Limit 22 to English language | **287** |
| Embase | | |
| 1 | exp JUVENILE RHEUMATOID ARTHRITIS/ di, dm, dr, dt, ep, rh, su, th | 6697 |
| 2 | JIA | 4807 |
| 3 | CHILD* or ADOLESCENT | 2630948 |
| 4 | ARTHRITIS/ di, dm, dr, dt, ep, rh, su, th | 16033 |
| 5 | 3 and 4 | 1546 |
| 6 | 1 or 2 or 5 | 11574 |
| 7 | exp REMISSION | 88438 |
| 8 | DISEASE ACTIVITY | 53363 |
| 9 | LOW or MINIMAL or ABSEN* or QUIESCE* | 2908940 |
| 10 | 8 and 9 | 11304 |
| 11 | DISEASE OUTCOME* | 8630 |
| 12 | INACTIVE DISEASE | 1910 |
| 13 | ACCEPTABLE SYMPTOM STATE | 115 |
| 14 | 7 or 10 or 11 or 12 or 13 | 107149 |
| 15 | 6 and 15 | 1259 |
| 16 | Limit 15 to (CLINICAL TRIAL or RANDOMIZED CONTOLLED TRIAL or CONTROLLED CLINICAL TRIAL or PHASE 1 CLINICAL TRIAL or PHASE 2 CLINICAL TRIAL or PHASE 3 CLINICAL TRIAL or PHASE 4 CLINICAL TRIAL) | 154 |
| 17 | Limit 15 to (CONFERENCE ABSTRACT or CONFERENCE PAPER or CONFERENCE PROCEEDING or CONFERENCE REVIEW) | 554 |
| 18 | Limit 15 to (META ANALYSIS or SYSTEMATIC REVIEW) | 13 |
| 19 | 15 not (16 or 17 or 18) | 578 |
| 20 | Limit 19 to English language | **531** |
| Pubmed | | |
| 1 | JUVENILE or CHILD* or ADOLESCENT | 3025294 |
| 2 | ARTHRITIS or POLYARTHRIT* or OLIGOARTHRIT* OR STILL’S | 263312 |
| 3 | 1 AND 2 | 11365 |
| 4 | REMISSION | 117611 |
| 5 | INACTIVE DISEASE | 10333 |
| 6 | DISEASE ACTIVITY and (LOW or MINIMAL or ABSEN* or QUIESCE*) | 55572 |
| 7 | DISEASE OUTCOME* | 6623 |
| 8 | ACCEPTABLE SYMPTOM STATE | 128 |
| 9 | 4 or 5 or 6 or 7 or 8 | 186094 |
| 10 | 3 and 9 | 1637 |
| 11 | Limit 10 to Journal Article | 1602 |
| 12 | Limit 11 to publication after 01/01/2013 | 272 |
| 13 | Limit 12 to English Language | 262 |
| 14 | Limit 13 to not medline[sb] | **74** |

**Supplementary Table 2.** The Quality Assessment (QA) Tool adapted from Pasma and others [18] and the Cochrane Collaboration tool for assessing risk of bias [19] to assess bias in selected articles

| Measure of quality assessed | Coding framework | | |
| --- | --- | --- | --- |
| Appropriate methods to select participants |  |  |  |
| 1. Sampling frame, age and sex of sample described | Yes | No | Don’t know |
| 1. **>80% participation or comparison of consents and refusals** | Yes | No | Don’t know |
| Appropriate methods to measure remission |  |  |  |
| 1. **Measure of remission reproducible** | Yes | No | Don’t know |
| 1. Remission measure | Validated objective | Non-validated objective | Non-validated subjective |
| Appropriate methods to reduce bias in design or analysis |  |  |  |
| 1. **Serious selection bias reduced by consecutive or stratified sampling** | Yes | No | Don’t know |
| 1. Proportion of patients in remission was a primary outcome | Yes | No | Don’t know |
| 1. **Serious bias arising from missing data reduced by adhering to at least one of the following:**  - No missing remission data - Reason for missing data likely unrelated to outcome - The proportion of missing remission data not enough to have a clinically relevant impact on results - Missing data imputed using appropriate methods   **Criteria for ‘don’t know’:**   - Numbers censored or with incomplete remission data not reported | Yes | No | Don’t know |
| Conflict of interest |  |  |  |
| 1. Conflict of interest declaration | Yes | No | Don’t know |

Questions in bold refer to ‘essential’ items.

Supplementary Table 3. Full results from the quality assessment tool for all selected articles

| Study | 1 | **2 (E)** | **3 (E)** | 4 | **5 (E)** | 6 | **7 (E)** | 8 | Total: | **Total essentials** | **Overall article quality** |
| --- | --- | --- | --- | --- | --- | --- | --- | --- | --- | --- | --- |
| Guzman et al., 2014 [22] | Yes | **Yes** | **Yes** | Non-validated objective | **Yes** | Yes | **Yes** | Yes | 7 | **4** | **High** |
| Berntson et al., 2014 [23] | Yes | **Yes** | **Yes** | Validated objective | **No** | No | **Yes** | Yes | 6 | **3** | **High** |
| Berntson et al., 2013 [24] | Yes | **Don’t know** | **Yes** | Validated objective | **Yes** | Yes | **No** | Yes | 6 | **2** | **Low** |
| Shen et al., 2013 [25] | Yes | **Yes** | **Yes** | Validated objective | **Yes** | No | **Yes** | Yes | 7 | **4** | **High** |
| Shen et al., 2013 [20] | Yes | **Don’t know** | **Yes** | Validated objective | **Yes** | Yes | **No** | Yes | 6 | **2** | **Low** |
| Nordal et al., 2011 [28] | Yes | **Don’t know** | **Yes** | Validated objective | **Yes** | Yes | **No** | Yes | 6 | **2** | **Low** |
| Oen et al., 2009 [29] | Yes | **Yes** | **Yes** | Non-validated objective | **Yes** | Yes | **No** | Yes | 6 | **3** | **High** |
| Berntson et al., 2007[30] | Yes | **Don’t know** | **Yes** | Non-validated objective | **Don’t know** | No | **No** | No | 2 | **1** | **Low** |
| Gäre et al., 1993 {Gare, 1993 1988 /id} | Yes | **Don’t know** | **Yes** | Non-validated objective | **Yes** | Don’t know | **No** | No | 3 | **2** | **Low** |
| Bertilsson et al., 2013 [26] | Yes | **Yes** | **Yes** | Non-validated objective | **Yes** | Yes | **No** | Yes | 6 | **3** | **High** |
| Bertilsson et al., 2012 [27] | Yes | **Yes** | **No** | Non-validated subjective | **Yes** | Yes | **No** | Yes | 5 | **2** | **Low** |
| Flatø et al., 1998 [36] | Yes | **Yes** | **Yes** | Non-validated objective | **Yes** | Yes | **No** | Yes | 6 | **3** | **High** |
| Gäre et al., 1995 [31] | Yes | **Yes** | **Yes** | Non-validated objective | **Yes** | No | **No** | Yes | 5 | **3** | **High** |
| Gäre et al., 1995 [32] | Yes | **Yes** | **Yes** | Non-validated objective | **Yes** | No | **No** | No | 4 | **3** | **Low** |
| Padeh et al., 2013 [34] | Yes | **Don’t know** | **Yes** | Validated objective | **Yes** | Yes | **No** | No | 5 | **2** | **Low** |
| Selvaag et al., 2006 [35] | Yes | **Yes** | **Yes** | Non-validated objective | **Yes** | Yes | **No** | Yes | 6 | **3** | **High** |
| Kotaniemi et al., 2002 [21] | Yes | **Yes** | **Yes** | Non-validated objective | **Yes** | Yes | **No** | No | 5 | **3** | **High** |

E: Essential items. For column 4, one point is awarded where validated objective criteria was implemented. For all other columns, only an answer of ‘Yes’ scores one point. A high quality article was defined as scoring ‘yes’ on at least three of the four essential questions or scoring at least five points overall.

Supplementary Table 4. The frequencies of remission across ILAR subtypes in selected articles

| **Author** | **Remission criteria** | **Disease duration at assessment** | **When remission assessed** | **Entire cohort** | | **Percent in remission within disease subtypes** | | | | | | | |
| --- | --- | --- | --- | --- | --- | --- | --- | --- | --- | --- | --- | --- | --- |
|  |  |  |  | **Percent ever remission (%)** | **Percent current remission (%)** | **Systemic** | **Oligo** | **RF- Poly** | **RF+ Poly** | **Poly in general** | **ERA** | **PsA** | **U.** |
| **Multi-centre** | | | | | | | | | | | | | |
| Guzman et al., 2014 [22] | Investigator defined ID and CR | 5 years | Throughout follow-up | 1 year:  45 (ID)  2 yrs:  78 (ID)  4 (CR)  3 yrs:  85 (ID)  13 (CR)  4 yrs:  92 (ID)  28 (CR)  5 yrs:  95 (ID)  41 (CR) | - | 1yr:  45 (ID)  2yrs:  71 (ID)  4.8 (CR)  3yrs:  73 (ID)  11 (CR)  4yrs:  85 (ID)  29 (CR)  5yrs:  85 (ID)  47 (CR) | 1yr:  61 (ID)  2yrs:  86 (ID)  7.6 (CR)  3yrs:  92 (ID)  21 (CR)  4ys:  96 (ID)  41 (CR)  5yrs:  96 (ID)  58 (CR) | 1yr:  34 (ID)  2yrs:  71 (ID)  1.1 (CR)  3yrs:  78 (ID)  3.2 (CR)  4yrs:  88 (ID)  7.7 (CR)  5yrs:  97 (ID)  14 (CR) | 1yr:  22 (ID)  2yrs:  48 (ID)  0 (CR)  3yrs:  67 (ID)  0 (CR)  4yrs:  79 (ID)  0 (CR)  5yrs:  93 (ID)  0 (CR) | - | 1yr:  34 (ID)  2yrs:  72 (ID)  1.9 (CR)  3yrs:  87 (ID)  5.8 (CR)  4yrs:  92 (ID)  28 (CR)  5yrs:  93 (ID)  47 (CR) | 1yr:  46 (ID)  2yrs:  91 (ID)  7.2 (CR)  3yrs:  93 (ID)  21 (CR)  4yrs:  92 (ID)  47 (CR)  5yrs:  100 (ID)  47 (CR) | 1yr:  33 (ID)  2yrs:  78 (ID)  1.2 (CR)  3yrs:  84 (ID)  11 (CR)  4yrs:  89 (ID)  30 (CR)  5yrs:  100 (ID)  46 (CR) |
| Berntson et al., 2014 [23] | Wallace’s preliminary criteria | 8 years | End of follow-up | - | 67 (ID) | - | - | - | - | - | - | - | - |
| Berntson et al., 2013 [24] | Wallace’s preliminary criteria | 8 years | End of follow-up | - | 41 (CR) | - | - | - | - | - | - | - | - |
| Shen et al., 2013 [25] | Wallace’s preliminary criteria | Median 8.7 years  (IQR 6.0 to 12.5) | Throughout and end of follow-up | 52 (CR) | 15 (CRM)  45 (CR) | End of follow-up:  14 (CRM)  54 (CR)  Throughout:  50 (CRM) | End of follow-up:  Pers: 19 (CRM)  66 (CR)  Ext: 23 (CRM)  39 (CR)  Throughout:  Pers:  69 (CR)  Ext:  62 (CR) | End of follow-up:  13 (CRM)  48 (CR)  Throughout:  48 (CR) | End of follow-up:  33 (CRM)  11 (CR)  Throughout:  11 (CR) | - | End of follow-up:  11 (CRM)  33 (CR)  Throughout:  41 (CR) | End of follow-up:  33 (CRM)  67 (CR)  Throughout:  67 (CR) | End of follow-up:  15 (CRM)  45 (CR)  Throughout:  28 (CR) |
| Bertilsson et al., 2013 [26] | Investigator defined ID and CR | 5 years  17 years | At 5 and 17 year follow-up | - | 5 years:  20 (ID)  38 (CR)  17 years:  19 (ID)  40 (CR) | 33 (ID)  50 (CR) | 5yrs:  18 (ID)  44 (CR)  17yrs:  31 (ID)  43 (CR) | - | - | 5yrs:  24 (ID)  24 (CR)  17yrs:  6 (ID)  39 (CR) | 5yrs:  0 (ID)  0 (CR)  17yrs:  0 (ID)  17 (CR) | 5yrs:  0 (ID)  25 (CR)  17yrs:  25 (ID)  25 (CR) | - |
| Shen et al., 2013 [20] | Wallace’s preliminary criteria | 1.5 years | End of follow-up | - | 53 (ID) | 67 (ID) | 90 (ID) | - | - | 44 (ID) | 39 (ID) | - | 23 (MDA)  47 (CR) |
| Bertilsson et al., 2012 [27] | Investigator defined ID and CR | 5 years | End of follow-up | - | 25 (ID)  34 (CR) | - | 28 (ID)  39 (CR) | - | - | 21 (ID)  24 (CR) | 0 (ID)  0 (CR) | 0 (ID)  50 (CR) | - |
| Nordal et al., 2011 [28] | Wallace’s preliminary criteria  PGA=0  PGE=0 | Median 8.2 years (IQR 7.0 to 12.3) | End of follow-up | - | 54 (PGA=0)  50 (PGE=0)  9 (CRM)  42 (CR) | 75 (PGA=0)  77 (PGE=0)  0 (CRM)  83 (CR) | Pers:  73 (PGA=0)  74 (PGE=0)  3.2 (CRM)  66 (CR)  Ext:  41 (PGA=0)  33 (PGE=0)  16 (CRM)  21 (CR) | 46 (PGA=0)  39 (PGE=0)  14 (CRM)  28 (CR) | 33 (PGA=0)  33 (PGE=0)  0 (CRM)  33 (CR) | - | 42 (PGA=0)  39 (PGE=0)  8.2 (CRM)  31 (CR) | 58 (PGA=0)  62 (PGE=0)  23 (CRM)  23 (CR) | 49 (PGA=0)  37 (PGE=0)  6.3 (CRM)  41 (CR) |
| Oen et al., 2009 [29] | Wallace’s preliminary criteria | Mean 6 months | End of follow-up | - | 33 (ID) | 27 (ID) | 46 (ID) | 19 (ID) | 8 (ID) | - | 19 (ID) | 35 (ID) | 32 (ID) |
| Berntson et al., 2007 [30] | No active joints  PGA=0  PGE=0 | 8 years | A random point throughout follow-up | - | 45 (No active joints)  20 (PGA=0)  35 (PGE=0) | - | - | - | - | - | - | - | - |
| Gäre et al., 1995 [31] | Investigator defined ID and CR | Median 7 years (range 1.5 to 21.9) | End of follow-up | - | 20 (ID)  31 (CR) | 5yrs:  25 (ID)  75 (CR)  17yrs:  25 (ID)  75 (CR) | 52 (Pers; CR) | - | - | 48 (ID) | 40 (ID) | 0 (ID) | - |
| Gäre et al., 1995 [32] | No active joints | Median 7 years  (range 1.5 to 21.9) | End of follow-up | - | 37 | - | - | - | - | - | - | - | - |
| Gäre et al., 1993 {Gare, 1993 1988 /id} | Investigator defined ID and CR | Group 1: mean 8.1 years (SD: 4.4)  Group 2: Mean 5 years (SD: 0.5) | End of follow-up | - | Group 1:  25 (ID)  37 (CR)  Group 2:  31 (ID)  43 (CR) | - | - | - | - | - | - | - | - |
| **Single centre** | | | | | | | | | | | | | |
| Padeh et al., 2013 [34] | Wallace’s preliminary criteria | Mean 1.5 years (SD: 0.5) | End of follow-up | - | 7 (CR) | - | - | - | - | - | - | - | - |
| Selvaag et al., 2006 [35] | Investigator defined CR | Mean 3.2 years (SD: 0.4) | End of follow-up | - | 26 | 29 | 39 (Pers)  6 (Ext) | 16 | 0 | - | 0 | 20 | - |
| Kotaniemi et al., 2002 [21] | Investigator defined CR | Mean 4.5 years | End of follow-up | - | 37 | - | - | - | - | - | - | - | - |
| Flatø et al., 1998 [36] | ACR 1981 RA remission criteria (ID) sustained for ≥6 months (CR) | Mean 9.7 years (SD: 2.1) | End of follow-up | - | 60 (ID)  47 (CR) | 0 | 84 (Pers)  28 (Ext) | - | - | 65 | 33 | 71 | - |

Oligo: Oligoarticular JIA; RF- Poly: Rheumatoid factor negative polyarticular JIA; RF+ Poly: Rheumatoid factor positive polyarticular JIA; ERA: Enthesitis-related JIA; PsA: Psoriatic JIA, U: Undifferentiated JIA; PGA: Physician’s global assessment of disease activity; PGE: Parental global assessment of disease activity; ESR: Erythrocyte sedimentation rate; CRP: C-reactive protein; ACR: American College of Rheumatology; RA: Rheumatoid arthritis; CR: Clinical remission off medication; CRM: Clinical remission on medication, SD: Standard deviation, IQR: Interquartile range.
